# Inter-operator reliability of the total decomposition score (TDS) method for estimating the post-mortem interval (PMI) in outdoor cases

**DOI:** 10.1007/s00414-025-03681-1

**Published:** 2025-11-28

**Authors:** Valentina Bugelli, Michele Strocchi, Tommaso Filippini, Anna Laura Santunione, Francesco Calabrò, Rossana Cecchi

**Affiliations:** 1 Medicine and Surgery Department, Forensic Pathology Section, University of Parma, Parma, Italy; 2 Department of Legal Medicine, AUSL Romagna, Cesena, Italy; 3 CREAGEN - Environmental, Genetic and Nutritional Epidemiology Research Center, Section of Public Health, Department of Biomedical, Metabolic and Neural Sciences, University of Modena and Reggio Emilia, Modena, Italy; 4 Institute of Legal Medicine, Department of Biomedical, Metabolic and Neural Sciences, University of Modena and Reggio Emilia, Modena, Italy

**Keywords:** Decomposition, Forensic science, Forensic taphonomy, Post-mortem interval, Total decomposition score

## Abstract

In the estimation of the Post-Mortem Interval (PMI), semi-quantitative methods have been proposed to overcome the challenges associated with determining the time of death. Among these, the Total Decomposition Score (TDS) method, developed by Gelderman et al., offers a systematic and semi-quantitative approach for estimating PMI. The aim of this study was to evaluate the reliability of the TDS by assessing its interoperator variability and comparing the results obtained with known reference data. A TDS-based questionnaire was administered to 100 participants - including forensic pathologists, residents in forensic medicine and professionals in forensic thanatology - using a dataset of six outdoor cadavers representing different decomposition stages. Data were analyzed using Fleiss’ Kappa (K) to assess inter-rater agreement and Spearman’s rank correlation to evaluate consistency. The results showed moderate overall agreement, with inter-rater reliability decreasing in cases with PMI exceeding 30 days. Linear regression analyses between estimated and actual post-mortem intervals yielded low coefficients of determination, with R² = 34.1% for the TDS-based model and R² = 20.5% for the ADD-based model, indicating that both methods explain only a limited portion of the variance in the actual PMI (PMIa). No statistically significant differences were observed among the professional categories, supporting the method’s applicability across different levels of expertise. While TDS shows promise as a practical tool for PMI estimation in field conditions, inter-operator variability remains a limiting factor in advanced decomposition stages.

**Supplementary Information:**

The online version contains supplementary material available at 10.1007/s00414-025-03681-1.

## Introduction

The estimation of the PMI remains one of the most complex challenges in forensic medicine, as it is influenced by a multitude of environmental and biological factors [1–4]. Over the years, various methods have been developed to enhance the accuracy of PMI estimation, based on physical, chemical and biological indicators. Among these, the analysis of human body decomposition represents a widely utilized approach, as it provides a direct indication of the time elapsed since death [5, 6].

Galloway et al. [7] introduced a structured decomposition classification, an approach that was subsequently revisited and further developed by various authors, often relying on qualitative and experience-based observations [8–10]. Following death, the body undergoes profound biochemical changes, including oxygen depletion, inhibition of enzymatic reactions, depletion of metabolites and cellular disintegration. These biological transformations, when studied under controlled conditions, have enabled the development of increasingly refined tools for PMI estimation—most notably, semi-quantitative methods that serve as a bridge between purely observational and fully quantitative approaches [11].

Semi-quantitative methods for PMI estimation bridge the gap between traditional observational techniques and fully quantitative approaches. Semi-quantitative methods, which combine observable decomposition characteristics with measurable environmental factors, have been critical in advancing the PMI estimation field.

In recent decades, the development of semi-quantitative methodologies has significantly improved the accuracy of PMI estimation by integrating intrinsic and extrinsic decomposition variables. Megyesi et al. [9] introduced the TBS, a decomposition classification system that, in combination with the Accumulated Degree-Days (ADD) model, enabled the estimation of PMI based on observable macroscopic changes in decomposed human remains. Subsequently, TBS was adapted for application in aquatic environments, leading to the development of the Total Aquatic Decomposition Score (TADS) (Heaton et al.) [12], designed to account for the specific decomposition processes occurring in water.

In parallel, Vass [13] proposed a decomposition model based on chemical variables, formulating predictive equations for both aerobic (surface) and anaerobic (buried) contexts. His work emphasized the critical influence of temperature, humidity and oxygen availability on decomposition kinetics. Additionally, research conducted in South Africa by Myburgh et al. [10] highlighted the necessity of regional calibration of decomposition models, reinforcing the role of climatic context in enhancing accuracy and applicability.

Among the most recent contributions to the field, the TDS represents a further step toward the standardization of decomposition assessment [14]. This semi-quantitative method is based on the evaluation of specific macroscopic decomposition parameters, allowing for the assignment of a standardized score to each stage of the process. The method separates the body into three anatomical regions—face and neck, torso and limbs—each of which decomposes differently and is therefore scored independently. Each region is evaluated using a scale of six stages, ranging from 1 (no visible decomposition) to 6 (complete skeletonization). Although each stage includes characteristic decomposition phenomena, these are not scored individually, since multiple changes may occur simultaneously or in varying order. Evaluators assign scores based on standardized descriptions provided by the authors for each decomposition stage, which are intended to guide consistent application of the method across different observers.

This study aims to evaluate the reliability of the TDS method by assessing the agreement among different forensic medicine specialists and practitioners to determine its reproducibility and potential application in PMI estimation.

## Materials and methods

### Study design

This study was designed as a diagnostic test accuracy investigation to assess the inter-operator reliability of the TDS method. A structured questionnaire was developed to collect both quantitative and qualitative data from forensic professionals, allowing for the analysis of variability, consistency and reliability in the application of the TDS.

### Case selection

The questionnaire was developed using six cadaveric cases recovered from outdoor environments in the Tuscany region (Köppen-Geiger classification: Csa) [15] between 2019 and 2022. Case selection followed the methodological inclusion and exclusion criteria established by Gelderman et al. [14], to ensure consistency with the original study design and comparability of findings. Inclusion criteria comprised adult individuals (>18 years) with complete and photographically documented remains and a reliably established PMI derived from multiple converging sources. Exclusion criteria included cases involving submerged, thermally altered or buried remains; individuals deceased in institutional healthcare settings; individuals under the age of 18; and cases with complete skeletonization (TDS = 18), in which decomposition assessment based on soft tissue was no longer feasible [13].

As a result, six cadaveric cases were included in the present analysis, all involving Caucasian adults between 49 and 82 years of age, with complete remains and a reliably established PMI.

Judicial autopsies and forensic entomological investigations were conducted for all included cases. Only cases with a reliably reconstructed PMI - based on a combination of autopsy findings, witness statements, media reports, last known contact and entomological analysis - were included. The PMIa, expressed in days, was derived from the integration of these multiple sources.

Among the six cases, five were female (83.3%) and one was male (16.7%). All individuals were discovered outdoors: three during summer, two in winter and one in autumn.

Regarding body coverage, one cadaver was found unclothed, four were clothed and one was partially wrapped in a sleeping bag. Entomological analyses were performed for all cases and insect activity used to estimate the minimum post-mortem interval (minPMI) [14, 16–19].

ADD were calculated using meteorological data from the nearest weather stations, prioritizing stations with temperature readings comparable to thermal measurements taken at the discovery sites. No cases recorded ambient temperatures below 0 °C during the relevant post-mortem period [20, 21].

### Questionnaire development

A questionnaire was developed using *REDCap* software, licensed by the University of Modena and Reggio Emilia. It was designed to collect anonymized participant information, including professional background and level of expertise, as well as to assess decomposition scoring based on standardized high-resolution images of cadavers.

**Fig. 1:**
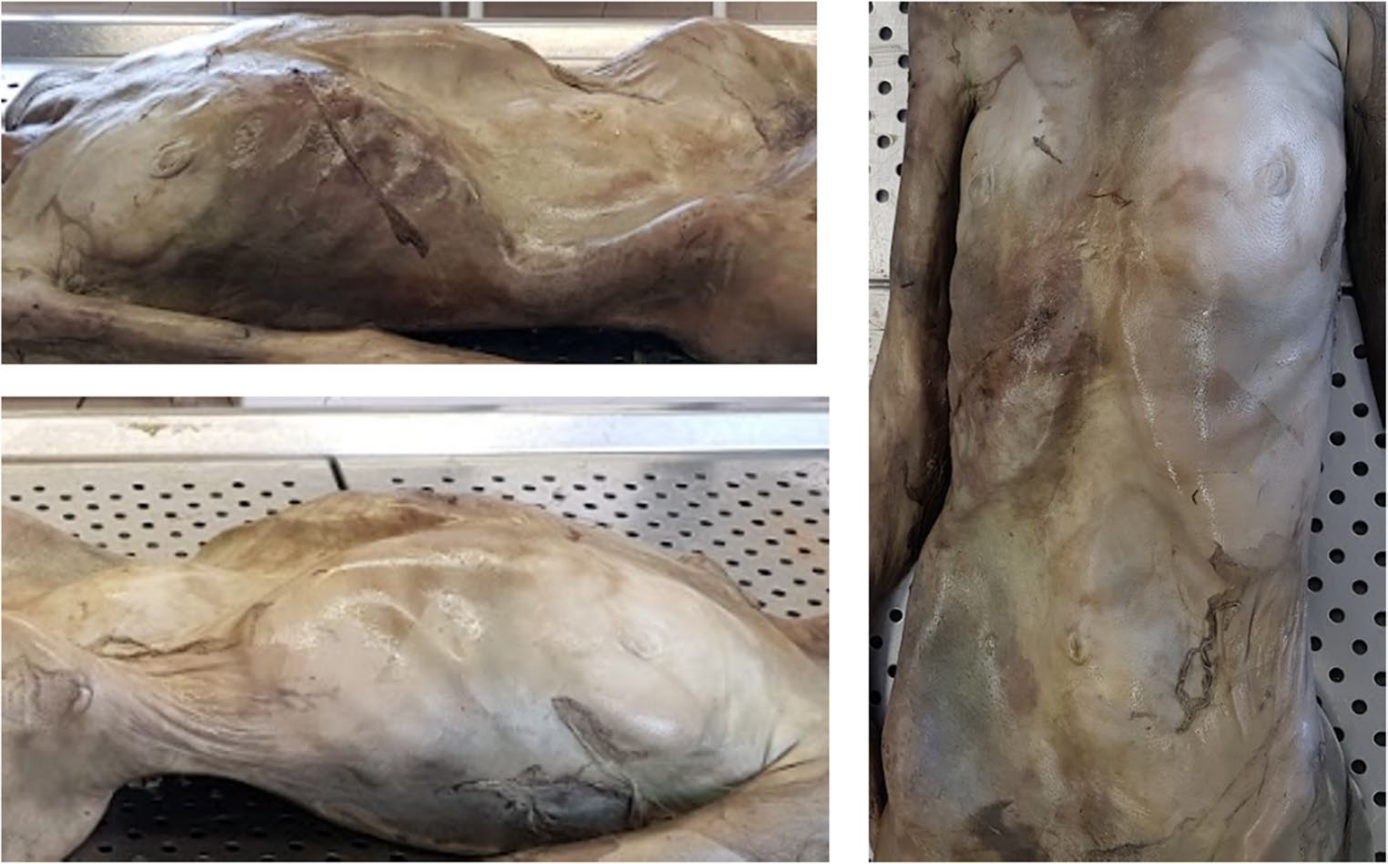
Example of an image illustrating body decomposition

**Fig. 2:**
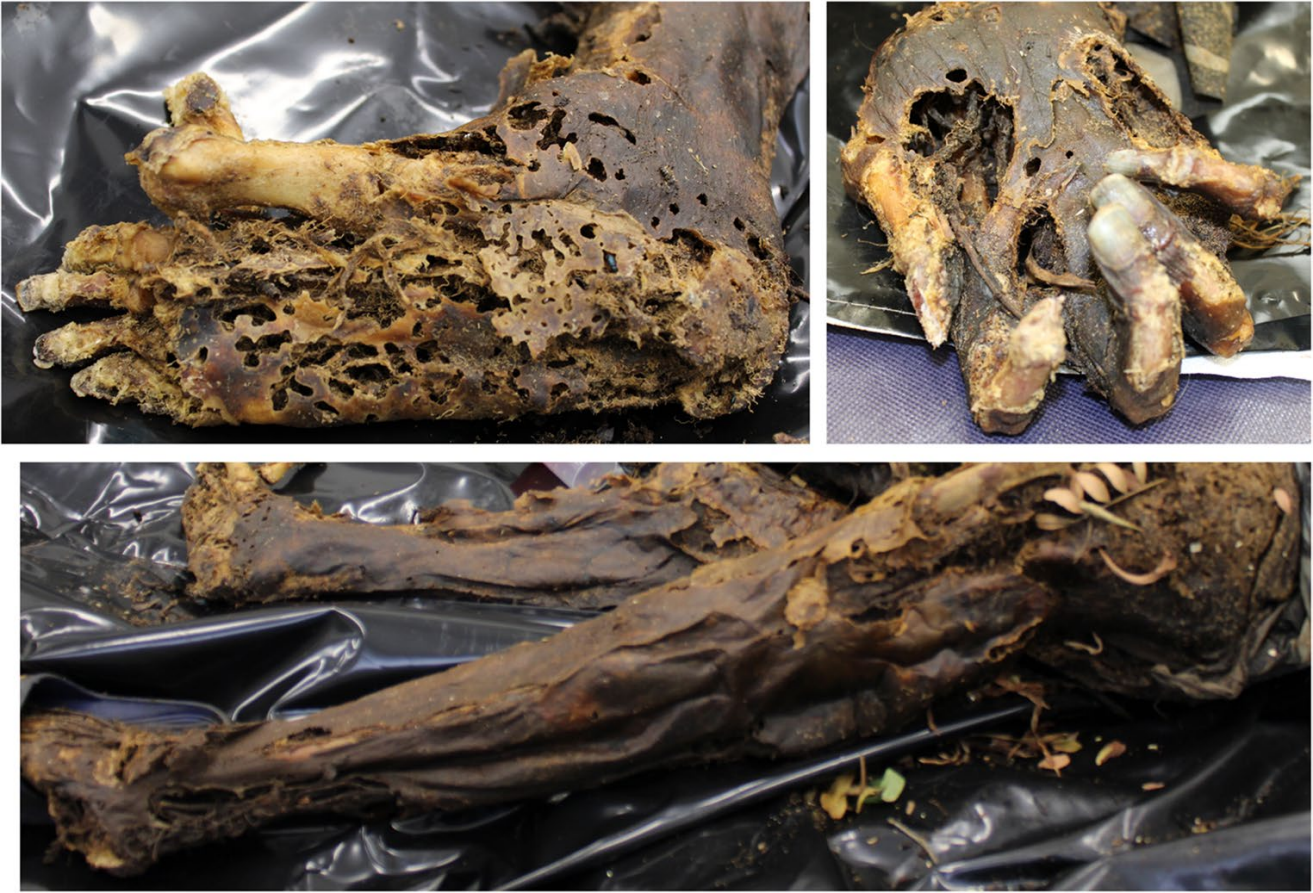
Example of an image illustrating limbs decomposition

Detailed instructions were provided to guide participants in assigning TDS scores to the presented cadaver images. The images were carefully selected to depict anatomical regions from multiple perspectives, following the model proposed in Gelderman et al.‘s book of reference [14] (Figs. 1 and 2).

The core of the questionnaire consisted of 18 randomized sections, each presenting images of the three anatomical regions for the six selected cases. Participants were asked to evaluate each image and assign a score using the TDS system based on their own assessment. The questionnaire administered to the participants is attached to the present article [attached link].

### Target population and sampling

The final questionnaire was distributed via a shared link through professional mailing lists and networks. Targeted recipients included forensic medicine specialists, residents in forensic medicine and other professionals such as forensic technicians and researchers. To ensure broad participation, the survey was also distributed through the National Council of Young Academic Forensic Pathologists (https://www.consultagiovanimedicilegali.it/). A target sample size of 100 participants was set to provide a robust dataset for statistical analysis.

### Statistical analysis

To evaluate the inter-operator variability in the application of the TDS method, statistical analyses were conducted using Fleiss’ Kappa (K) and Observed Agreement Proportion (P_i_).

Fleiss’ Kappa was applied to assess the level of agreement among multiple raters across categorical variables—specifically, the decomposition scores attributed to the facial (FDS), body (BDS) and limbs (LDS). Kappa values were interpreted according to the standard classification: values ≤ 0.20 indicate slight agreement, 0.21–0.40 fair, 0.41–0.60 moderate, 0.61–0.80 substantial and ≥ 0.81 almost perfect agreement [10]. The analysis was stratified by respondent groups (forensic medicine residents, specialists and other forensic professionals) and further differentiated based on autopsy experience among specialists.

To further explore the consistency of responses across individual cases, the P_i_ was analyzed for each anatomical region and each case included in the questionnaire. This index quantifies the proportion of raters who assigned the same score to a specific image, providing a detailed view of which scenarios showed greater or lesser consensus. For each case, rater-level PMI estimates were computed using both regression models proposed by Gelderman et al., namely the direct TDS-based model (PMI_(TDS)_) and the temperature-normalized model derived from accumulated degree-days (PMI_(ADD)_). Individual PMI_(TDS)_ and PMI_(ADD)_ values were obtained for each evaluator and case.

Correlations between the estimated PMI values (PMI_(TDS)_ and PMI_(ADD)_) and the PMIa were assessed using Spearman’s rank correlation coefficient (ρ), since the TDS is an ordinal variable and does not meet the assumptions of parametric correlation tests. Univariate linear regressions were used to assess the relation between PMIa and each of the two estimated PMI models (PMI_(TDS)_ and PMI_(ADD)_), and to evaluate the proportion of variance explained (R^2^). All statistical analyses were performed using Excel (Office Package, Microsoft Corp, 2025) and StataNow MP v19.5 (StataCorp LLC, College Station, TX, USA, 2025).

## Results

A total of 100 participants completed the questionnaire. Of these, 50 were forensic medicine residents (50%), 38 were specialists in forensic medicine (38%) and 12 were other professionals working in forensic thanatology (12%).

Among the specialists, 19 (50%) reported routinely performing autopsy procedures or medico-legal investigations, 17 (45%) did not and 2 (5%) did not provide a response. Within the autopsy-performing subgroup, 2 specialists (11%) had less than 5 years of experience, 7 (37%) had between 5 and 10 years and 10 (53%) had over 10 years of experience.

Fleiss’ Kappa was applied to assess inter-operator agreement. Across all participants, the values were 0.42 for the FDS, 0.38 for the BDS and 0.51 for the LDS. The highest agreement was recorded for LDS (K = 0.51) and the lowest for BDS (K = 0.38).

Within specific groups, residents achieved K values of 0.41 for FDS, 0.39 for BDS and 0.54 for LDS. Specialists showed similar agreement: 0.40 for FDS, 0.38 for BDS and 0.53for LDS. Other forensic professionals recorded values of 0.47 for FDS, 0.38 for BDS and 0.45 for LDS. In both the resident and specialist groups, LDS showed the highest agreement, whereas BDS had the lowest. Among other professionals, FDS was the most consistent (Table 1).

Further analysis among specialists based on autopsy experience revealed K values of 0.37 (FDS), 0.41 (BDS) and 0.47 (LDS) in those performing autopsies, and 0.44 (FDS), 0.33 (BDS) and 0.52 (LDS) in those without autopsy activity. Among specialists who perform autopsies, the highest agreement was observed for LDS and the lowest for FDS; among those who do not perform autopsies, LDS again showed the highest agreement, while BDS showed the lowest (Table 2).

The P_i_ were calculated for each of the six cadaver cases and anatomical regions.

For FDS, P_i_ ranged from 0.42 (Case 6) to 0.78 (Case 4), with intermediate values in Cases 1, 2, 3 and 5.

Regarding the BDS, P_i_ values spanned from 0.39 to 0.83. Case 4 again showed the highest agreement (0.83), followed by Case 3 (0.67) and Cases 2 and 6, both at 0.53. The lowest was observed in Case 1 (0.39), with Case 5 slightly higher at 0.44.

For the LDS, the observed agreement proportions ranged between 0.50 and 0.90. The peak was found in Case 4 (0.90), followed by Case 2 (0.85) and Case 3 (0.73). The lowest values appeared in Case 6 (0.50), with Cases 5 (0.53) and 1 (0.55) just above (Table 3).

**Table 1:** Results of the Fleiss kappa test for all groups of responders

	Forensic Medicine Residents	Forensic Medicine Specialists	Other Forensic Professionals	All responders
**FDS**	0.41	0.40	0.47	0.42
**BDS**	0.39	0.38	0.38	0.38
**LDS**	0.54	0.53	0.45	0.51

**Table 2:** Results of the Fleiss kappa test for forensic medicine specialists

	Forensic Medicine Specialists performing autopsy activities	Forensic Medicine Specialists not performing autopsy activities
**FDS**	0.37	0.44
**BDS**	0.41	0.33
**LDS**	0.47	0.52

**Table 3:** Observed agreement proportion (Pi) for all responders for every anatomical region

Cases	Pi for FDS	Pi for BDS	Pi for LDS
***1***	*0.55*	*0.39*	*0.55*
***2***	*0.58*	*0.53*	*0.85*
***3***	*0.69*	*0.67*	*0.73*
***4***	*0.78*	*0.83*	*0.90*
***5***	*0.50*	*0.44*	*0.53*
***6***	*0.42*	*0.53*	*0.50*

For each case, the average TDS score assigned by all respondents was calculated and subsequently used to estimate the PMI using the two regression models proposed by Gelderman et al. - one based directly on TDS (PMI_(TDS)_) and the other incorporating TDS-derived ADD (PMI_(ADD)_), obtained by dividing the ADD values derived from the regression formula by the corresponding average daily ambient temperature. For each case, the mean and standard deviation (SD) of the PMI estimates were calculated across all evaluators to quantify inter-operator variability. The mean inter-operator SDs across the six cases were 16.4 days for PMI_(TDS)_ and 13.1 days for PMI_(ADD)_, providing an indirect measure of the dispersion of the estimators. These estimated PMI values were then compared with the PMIa.

Table 4 reports, for each case, the mean and standard deviation of PMI estimates obtained from both the TDS-based and ADD-based models. The standard deviations of PMI_(TDS)_ ranged from 2.5 to 46.1 days (mean SD = 16.4), while those of PMI_(ADD)_ ranged from 1.4 to 45.6 days (mean SD = 13.1). These values reflect the variability among evaluators in assigning TDS scores and consequently in estimating PMI. Cases with longer post-mortem intervals (cases 1, 5, and 6) showed the largest SD values, confirming a progressive increase in inter-operator variability with advancing decomposition.

**Table 4:** Comparison of PMIa, expressed in days and PMI estimated using average TDS (PMI_(TDS)_) and PMI TDS-derived ADD (PMI_(ADD)_)

Cases	PMIa	PMI_(TDS)_ mean ± SD	PMI_(ADD)_ mean ± SD
**1**	61	50.2 ± 28.4	22.7 ± 13.7
**2**	6	9.8 ± 3.2	4.4 ± 1.5
**3**	30	7.7 ± 2.6	8.5 ± 3.0
**4**	8	5.6 ± 2.8	2.4 ± 1.4
**5**	36	84.8 ± 46.1	79.1 ± 45.6
**6**	56	27.2 ± 15.3	25.7 ± 15.4

Within the study, two comparisons were conducted regarding the estimation of the PMI using the method proposed by Gelderman. First, the PMI estimated directly using the TDS was compared to the PMIa. Second, the PMI estimated using Gelderman’s method, which incorporates the TDS for the calculation of ADD, was compared to the real PMI.

**Fig. 3:**
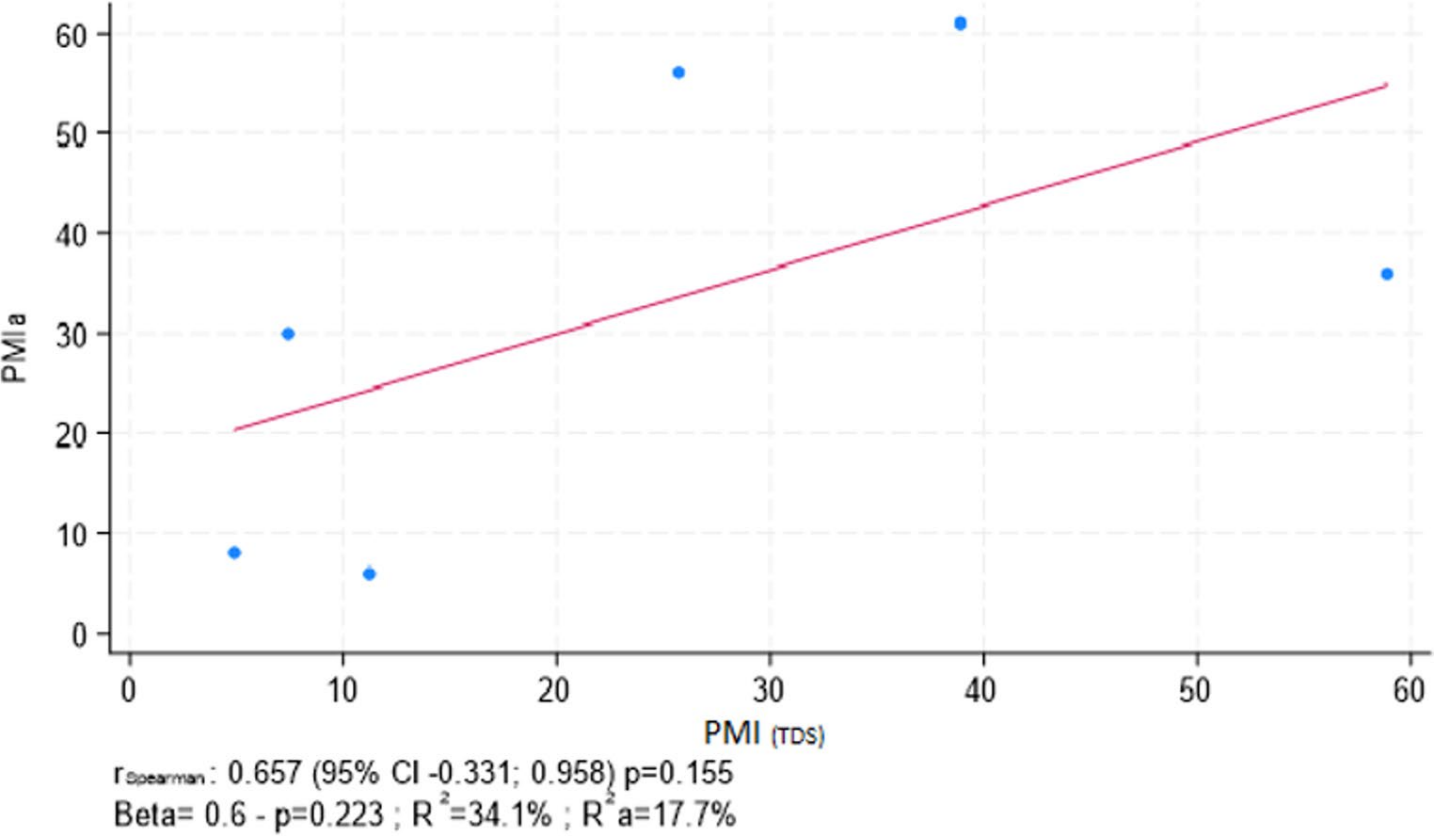
Scatter plot with correlation between PMI_(TDS)_ vs. PMIa. Coefficients from both Spearmen’s rank correlation (r_Spearman_) and linear regression analysis (Beta) are reported

**Fig. 4:**
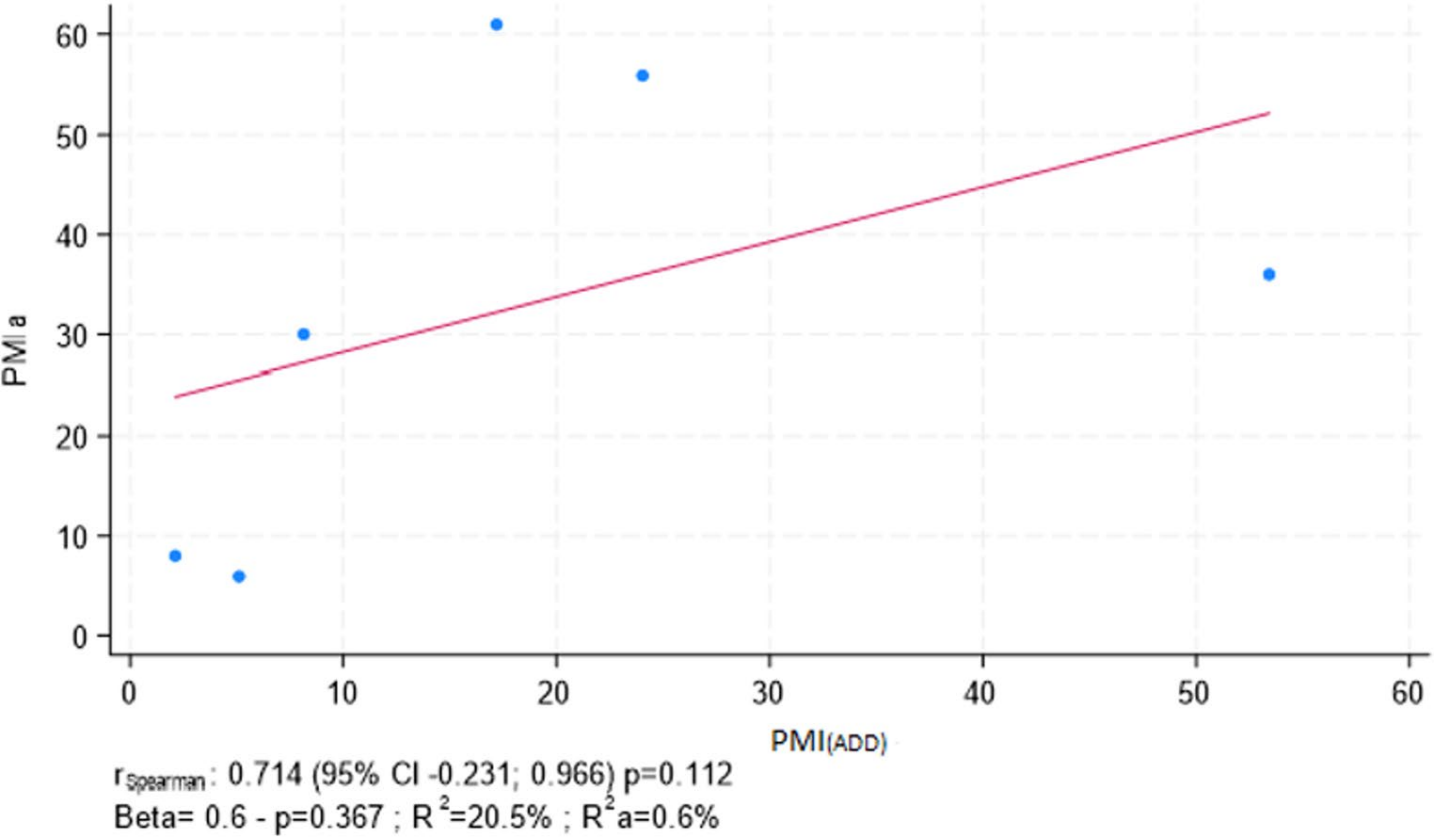
Scatter plot with correlation between PMI_(ADD)_ vs. PMIa. Coefficients from both Spearmen’s rang correlation (r_Spearman_) and linear regression analysis (Beta) are reported

PMI_(TDS)_ estimation showed a higher coefficient of determination compared to the PMIa, with a value of 34.1% (β = 0.6, *p* = 0.223) indicating better model performance than the PMI_(ADD)_ estimation, which had a lower R² value (20.5%, β = 0.6, *p* = 0.367) (Figs. 3 and 4).

## Discussion

The TDS, developed and validated by Gelderman et al. [14], represents a semi-quantitative framework for estimating the PMI and was designed to improve standardization and reproducibility in forensic investigations. Conceptually rooted on the TBS proposed by Megyesi et al. [9] and on Vass’s [13] approach, the TDS structure relies on the independent assessment of three anatomical regions—head and neck, torso and limbs—each evaluated across six decomposition stages defined by morphological criteria ranging from no visible changes to complete skeletonization. The terminology adopted in the TDS is consistent with that introduced by De Kat et al. [22] for aquatic decomposition scoring and adapted here for terrestrial settings as Facial (FDS), Body (BDS) and Limb (LDS) decomposition scores.

Unlike models based on isolated decomposition features, the TDS aggregates decomposition phenomena into broader stages, acknowledging that decomposition changes can occur simultaneously or in varying sequences. This flexible framework captures the intrinsic heterogeneity of the decomposition process, avoiding rigid chronological classifications.

The model incorporates two regression-based approaches for PMI estimation: one using the TDS value directly in a linear equation and the other converting TDS to ADD, which are then used to estimate PMI. Both methods assume consistent and accurate scoring, underscoring the importance of validating inter-observer reliability in the application of the system.

As with all semi-quantitative decomposition models, the TDS relies on narrative descriptors to guide scoring. This introduces a degree of subjectivity, which makes the clarity, precision and operational relevance of these descriptors essential. Consequently, the inter-operator consistency in applying these criteria is a critical factor in determining the method’s forensic reliability.

In this context, the extent to which the scoring system captures decomposition progression across a wide range of evaluators becomes central to its validation. While earlier decomposition scoring methods such as those by Megyesi and Vass did not systematically evaluate inter-rater variability, Gelderman et al. reported moderate to substantial agreement using Fleiss’ Kappa: twelve participants (four forensic physicians, four forensic scientists and four medical students), who had previously reviewed a standardized Book of Reference, independently scored 45 photographs. The lowest agreement was observed for facial scores assigned by medical students (κ = 0.74), while the highest was for body scores assigned by the same group (κ = 0.97). Overall, two scores showed moderate agreement and seven showed substantial to almost perfect agreement, confirming the method’s reliability across different user profiles [14].

In our study, among the anatomical regions, LDS achieved the highest agreement across evaluators (K = 0.51), particularly among forensic medicine specialists (K = 0.53) and residents (K = 0.54). The FDS followed with moderate agreement (K = 0.42), whereas the BDS yielded only fair agreement (K = 0.38). These results suggest that limb decomposition is easier to evaluate consistently, likely due to its simpler and more homogeneous visual characteristics, while the torso may present greater interpretative challenges due to more complex patterns of decomposition [23].

Compared to the original validation study by Gelderman et al., which reported higher levels of inter-observer agreement, our study yielded slightly lower values, ranging from fair to moderate.

Several factors may explain this discrepancy: the greater heterogeneity of the present sample, the absence of a structured preparatory phase involving the standardized descriptions provided by the authors and the selection of cases with a significantly higher average PMIa (32.8 days vs. 4.7 days in the Dutch study [14]). Environmental conditions affecting outdoor cases likely contributed to greater variability in decomposition patterns, impacting inter-rater consistency [4].

Notably, no substantial differences in agreement were found among the three evaluator categories. Even within the group of forensic medicine specialists, performing autopsy activity did not significantly affect concordance, with similar Kappa values observed regardless of autopsy experience. These findings are consistent with those of Gelderman et al., supporting the conclusion that the TDS method can be reliably applied by evaluators with different levels of experience.

The analysis of the P_i_ revealed higher concordance in cases where decomposition appeared to be in an intermediate phase, particularly in cases 2, 3 and 4. In these cases, most scores for FDS and BDS regions clustered around values 3 and 4, while scores for LDS were concentrated around 2, 3 and 4. This suggests that intermediate decomposition stages are characterized by clearer and more easily recognizable features, facilitating greater consistency among evaluators. Conversely, cases 1, 5 and 6—associated with more advanced decomposition—exhibited lower agreement, likely due to the increased complexity and subjectivity involved in interpreting decomposition features at later stages.

These trends were further supported by the PMIa of the cases. The highest levels of agreement were observed in cases with shorter PMIa (6, 30 and 8 days), while longer PMIa (61, 36 and 56 days) corresponded to lower proportions of agreement. This reinforces the notion that the stage of decomposition has a more substantial impact on inter-rater reliability than evaluator experience or training alone.

Additional considerations emerge from the comparison between the PMIa and the values estimated using the two formulas proposed by Gelderman et al. for outdoor cases. The analysis indicates that the formula which directly uses the TDS yields better predictive performance than the formula that incorporates ADD calculated from TDS. Specifically, the regression analyses revealed that both models explained a limited portion of the variance in the PMIa (R² = 34.1% for PMI_(TDS)_ and R² = 20.5% for PMI_(ADD)_). These findings confirm that, although the TDS-based model showed slightly better predictive performance, the decomposition-based estimation of PMI remains inherently uncertain. This limitation likely reflects the multifactorial nature of decomposition processes and the subjective variability in TDS scoring.

The analysis of standard deviations provided further insights into the reliability of both models. The inter-operator SDs were slightly higher for PMI(TDS) (mean = 16.4 days) than for PMI(ADD) (mean = 13.1 days), indicating marginally better consistency in the ADD-based model. This approach provided an indirect measure of the dispersion of the estimators. However, this difference did not translate into improved predictive accuracy, as shown by the lower coefficient of determination (R² = 20.5% for PMI(ADD) vs. 34.1% for PMI(TDS)). Therefore, while temperature normalization in the ADD model appears to reduce the internal dispersion of estimates, the direct TDS-based approach retains a stronger, albeit weak, correlation with the actual post-mortem interval. Overall, both models are similarly affected by the subjective variability of decomposition scoring, which remains a major limiting factor in PMI estimation.

These results could be particularly important for forensic experts applying the TDS method or being confronted with its results in court, as they highlight the need to interpret decomposition-based PMI estimates with caution and to clearly communicate their inherent uncertainty in judicial contexts.

This finding aligns with the conclusions of the original Dutch study, reinforcing the notion that in outdoor settings, where temperature and environmental conditions can fluctuate widely, the TDS-based estimation is more robust and less affected by climatic variability.

In outdoor environments, climate variability reduces the reliability of ADD-based estimates, while the direct use of TDS, less affected by external factors, provides a more stable and accurate assessment of decomposition.

Nevertheless, the analysis also revealed limitations, particularly in cases with extended PMI (> 30 days). In these instances, both formulas showed reduced precision, occasionally underestimating or overestimating the actual PMI. Conversely, in cases with shorter PMI (< 30 days), the estimates were generally more accurate and closely aligned with real values. This trend suggests a decrease in predictive accuracy as the TDS value increases, likely due to the plateauing of decomposition features in advanced stages.

## Conclusions

The findings support the utility of the TDS as a central and robust parameter for PMI estimation, as it consistently integrates decomposition features across anatomical regions and shows potential for reliable application even by less experienced users or in challenging conditions. Although both TDS- and ADD-based formulas proved effective, particularly in cases with shorter PMI, their predictive accuracy declined in cases of prolonged decomposition, emphasizing the need to consider PMI duration when applying these tools.

A key challenge identified was the high degree of inter-operator variability, especially in advanced decomposition stages. This highlights the importance of clearer scoring guidelines and more structured training resources, such as the standardized descriptions provided by the authors. Refining the scoring system—particularly through expanded categorization of late-stage decomposition—could reduce inconsistencies and enhance reproducibility among evaluators.

The comparison with the original validation study by Gelderman et al. also revealed methodological and contextual differences, including environmental conditions and sample composition, which further support the need for region-specific validation. In this context, expanding the dataset and incorporating a broader range of cases will be essential in future research to enhance the method’s generalizability.

## Supplementary Information

Below is the link to the electronic supplementary material.

Supplementary Material 1
