# Supplementary material for: Inter-operator reliability of the total decomposition score (TDS) method for estimating the post-mortem interval (PMI) in outdoor cases
Source: Int J Legal Med. 2025 Nov 28;140(2):1201–9. doi: 10.1007/s00414-025-03681-1 (PMC12957013; doi:10.1007/s00414-025-03681-1)
Supplement: Supplementary file 1 — Supplementary Material 1 [file 414_2025_3681_MOESM1_ESM.pdf]

1. Which stage of decomposition does image no. 1 represent?

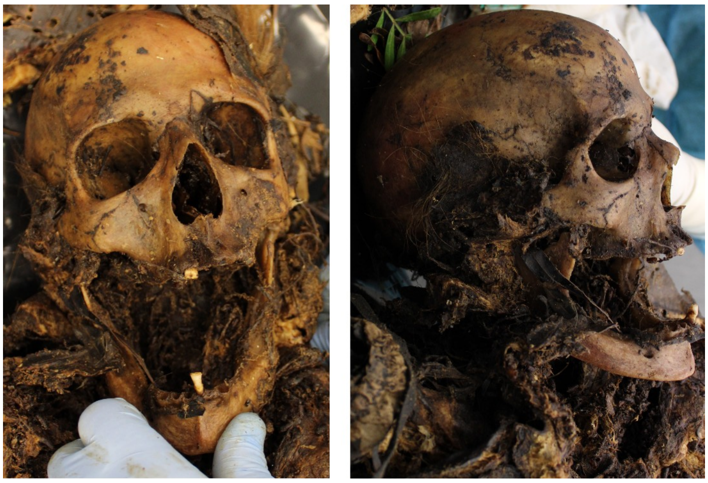

1 1.1 No visible changes
2 2.1 Livor mortis, rigour mortis and vibices
2.2 Eyes: cloudy and/or tache noir
2.3 Discoloration: brownish shades particularly at the edges. Drying of nose, ears and lips
3 3.1 Grey to green discoloration
3.2 Bloating of neck and face is present and/or skin blisters, skin slippage and/or marbling
3.3 Purging of decompositional fluids out of ears, nose and mouth and/or brown to black discoloration
4 4.1 Caving in of the flesh and tissues of eyes and throat. Skin having a leathery appearance
4.2 Partial skeletonization, joints still together
5 5.1 Gross skeletonization, some joints disarticulated
6 6.1 Complete skeletonization

2. Which stage of decomposition does image no. 2 represent?

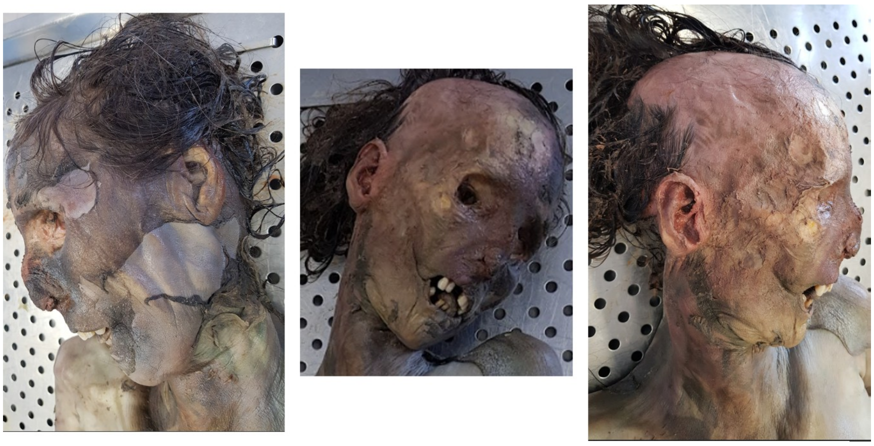

1 1.1 No visible changes
2 2.1 Livor mortis, rigour mortis and vibices
2.2 Eyes: cloudy and/or tache noir
2.3 Discoloration: brownish shades particularly at the edges. Drying of nose, ears and lips
3 3.1 Grey to green discoloration
3.2 Bloating of neck and face is present and/or skin blisters, skin slippage and/or marbling
3.3 Purging of decompositional fluids out of ears, nose and mouth and/or brown to black discoloration
4 4.1 Caving in of the flesh and tissues of eyes and throat. Skin having a leathery appearance
4.2 Partial skeletonization, joints still together
5 5.1 Gross skeletonization, some joints disarticulated
6 6.1 Complete skeletonization

3. Which stage of decomposition does image no. 3 represent?

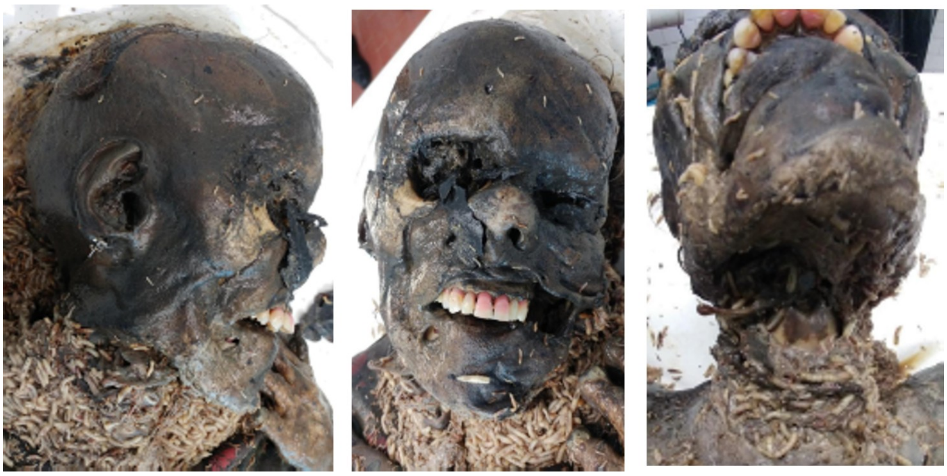

1	1.1 No visible changes
2	2.1 Livor mortis, rigour mortis and vibices
	2.2 Eyes: cloudy and/or tache noir
	2.3 Discoloration: brownish shades particularly at the edges. Drying of nose, ears and lips
3	3.1 Grey to green discoloration
	3.2 Bloating of neck and face is present and/or skin blisters, skin slippage and/or marbling
	3.3 Purging of decompositional fluids out of ears, nose and mouth and/or brown to black discoloration
4	4.1 Caving in of the flesh and tissues of eyes and throat. Skin having a leathery appearance
	4.2 Partial skeletonization, joints still together
5	5.1 Gross skeletonization, some joints disarticulated
6	6.1 Complete skeletonization

4. Which stage of decomposition does image no. 4 represent?

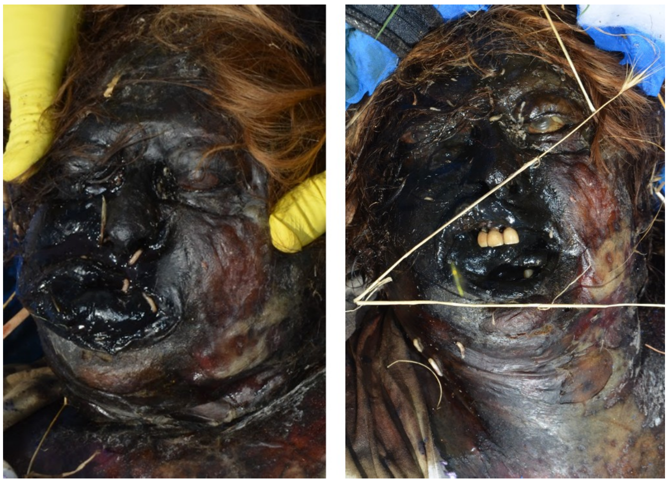

1	1.1 No visible changes
2	2.1 Livor mortis, rigour mortis and vibices
	2.2 Eyes: cloudy and/or tache noir
	2.3 Discoloration: brownish shades particularly at the edges. Drying of nose, ears and lips
3	3.1 Grey to green discoloration
	3.2 Bloating of neck and face is present and/or skin blisters, skin slippage and/or marbling
	3.3 Purging of decompositional fluids out of ears, nose and mouth and/or brown to black discoloration
4	4.1 Caving in of the flesh and tissues of eyes and throat. Skin having a leathery appearance
	4.2 Partial skeletonization, joints still together
5	5.1 Gross skeletonization, some joints disarticulated
6	6.1 Complete skeletonization

5. Which stage of decomposition does image no. 5 represent?

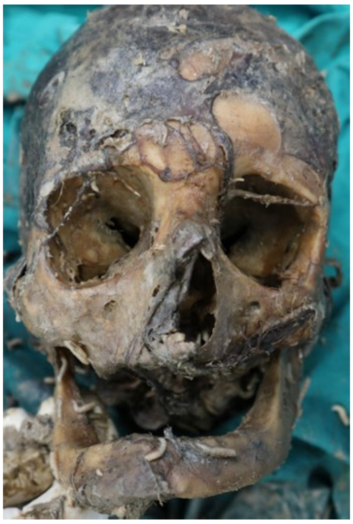

1 1.1 No visible changes

2 2.1 Livor mortis, rigour mortis and vibices

2.2 Eyes: cloudy and/or tache noir

2.3 Discoloration: brownish shades particularly at the edges. Drying of nose, ears and lips

3 3.1 Grey to green discoloration

3.2 Bloating of neck and face is present and/or skin blisters, skin slippage and/or marbling

3.3 Purging of decompositional fluids out of ears, nose and mouth and/or brown to black discoloration

4 4.1 Caving in of the flesh and tissues of eyes and throat. Skin having a leathery appearance

4.2 Partial skeletonization, joints still together

5 5.1 Gross skeletonization, some joints disarticulated

6 6.1 Complete skeletonization

6. Which stage of decomposition does image no. 6 represent?

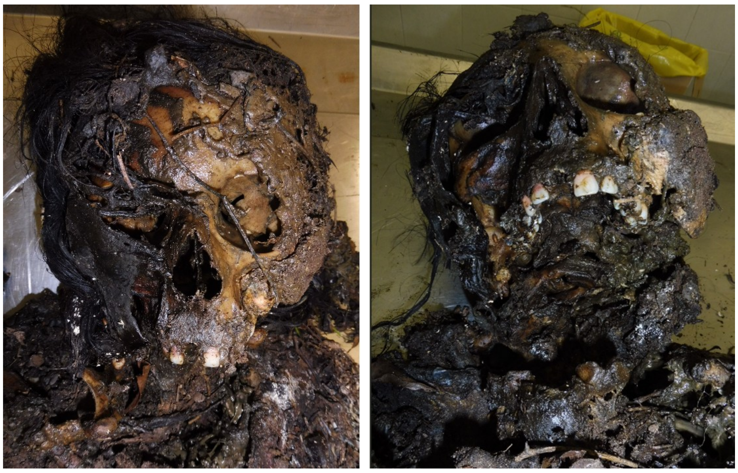

1 1.1 No visible changes

2 2.1 Livor mortis, rigour mortis and vibices

2.2 Eyes: cloudy and/or tache noir

2.3 Discoloration: brownish shades particularly at the edges. Drying of nose, ears and lips

3 3.1 Grey to green discoloration

3.2 Bloating of neck and face is present and/or skin blisters, skin slippage and/or marbling

3.3 Purging of decompositional fluids out of ears, nose and mouth and/or brown to black discoloration

4 4.1 Caving in of the flesh and tissues of eyes and throat. Skin having a leathery appearance

4.2 Partial skeletonization, joints still together

5 5.1 Gross skeletonization, some joints disarticulated

6 6.1 Complete skeletonization

7. Which stage of decomposition does image no. 7 represent?

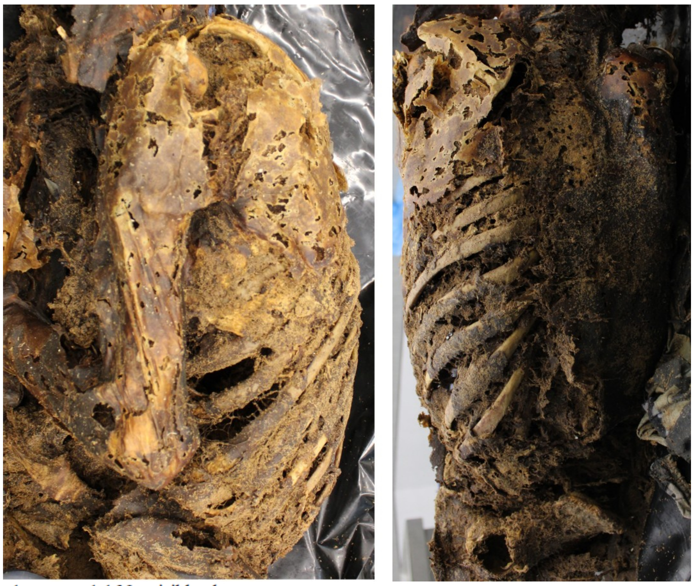

1 1.1 No visible changes
2 2.1 Livor mortis, rigour mortis and vibices
3 3.1 Grey to green discoloration
3.2 Bloating with green discoloration and/or skin blisters, skin slippage and/or marbling
3.3 Rectal purging of decompositional fluids
3.4 Post-bloating: release of abdominal gasses with discoloration changing from green to black
4 4.1 Decomposition of tissue producing sagging of flesh. Caving in of the abdominal cavity
4.2 Skin having a leathery appearance
4.3 Partial skeletonization, joints still together
5 5.1 Gross skeletonization, some joints disarticulated
6 6.1 Complete skeletonization

8. Which stage of decomposition does image no. 8 represent?

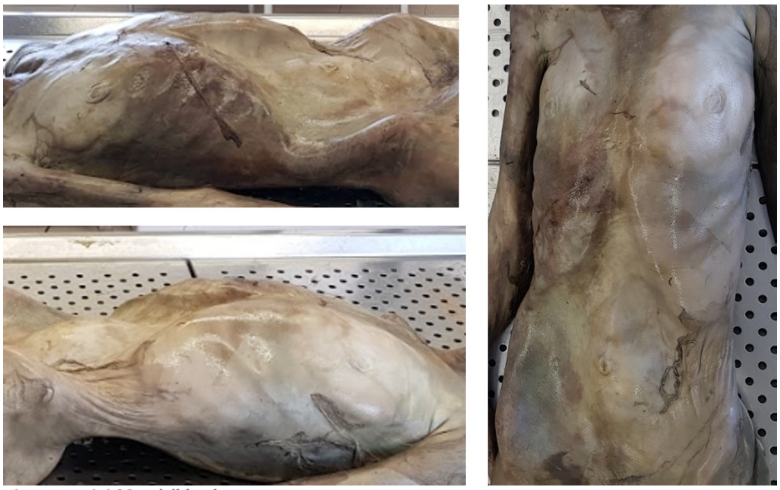

1 1.1 No visible changes
2 2.1 Livor mortis, rigour mortis and vibices
3 3.1 Grey to green discoloration
3.2 Bloating with green discoloration and/or skin blisters, skin slippage and/or marbling
3.3 Rectal purging of decompositional fluids
3.4 Post-bloating: release of abdominal gasses with discoloration changing from green to black
4 4.1 Decomposition of tissue producing sagging of flesh. Caving in of the abdominal cavity
4.2 Skin having a leathery appearance
4.3 Partial skeletonization, joints still together
5 5.1 Gross skeletonization, some joints disarticulated
6 6.1 Complete skeletonization

9. Which stage of decomposition does image no. 9 represent?

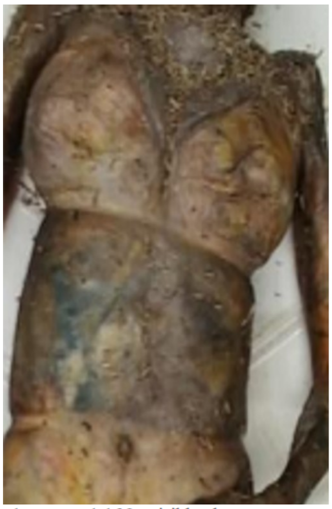

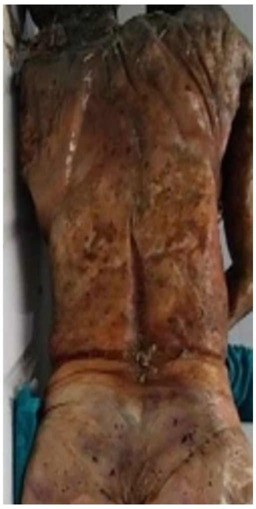

1	1.1 No visible changes
2	2.1 Livor mortis, rigour mortis and vibices
3	3.1 Grey to green discoloration
	3.2 Bloating with green discoloration and/or skin blisters, skin slippage and/or marbling
	3.3 Rectal purging of decompositional fluids
	3.4 Post-bloating: release of abdominal gasses with discoloration changing from green to black
4	4.1 Decomposition of tissue producing sagging of flesh. Caving in of the abdominal cavity
	4.2 Skin having a leathery appearance
	4.3 Partial skeletonization, joints still together
5	5.1 Gross skeletonization, some joints disarticulated
6	6.1 Complete skeletonization

10. Which stage of decomposition does image no. 10 represent?

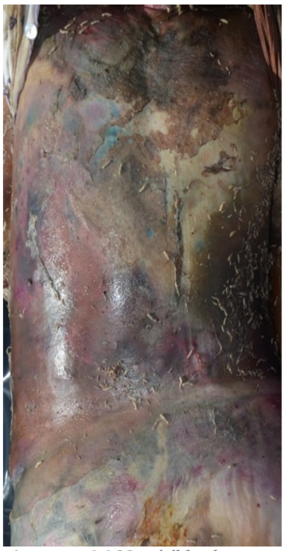

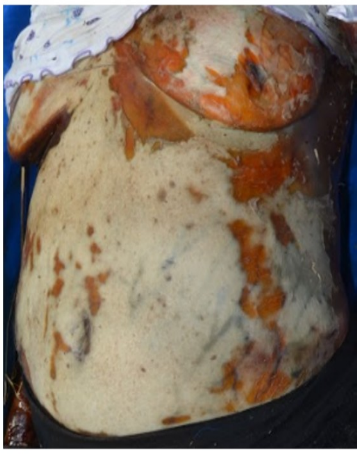

1	1.1 No visible changes
2	2.1 Livor mortis, rigour mortis and vibices
3	3.1 Grey to green discoloration
	3.2 Bloating with green discoloration and/or skin blisters, skin slippage and/or marbling
	3.3 Rectal purging of decompositional fluids
	3.4 Post-bloating: release of abdominal gasses with discoloration changing from green to black
4	4.1 Decomposition of tissue producing sagging of flesh. Caving in of the abdominal cavity
	4.2 Skin having a leathery appearance
	4.3 Partial skeletonization, joints still together
5	5.1 Gross skeletonization, some joints disarticulated
6	6.1 Complete skeletonization

11. Which stage of decomposition does image no. 11 represent?

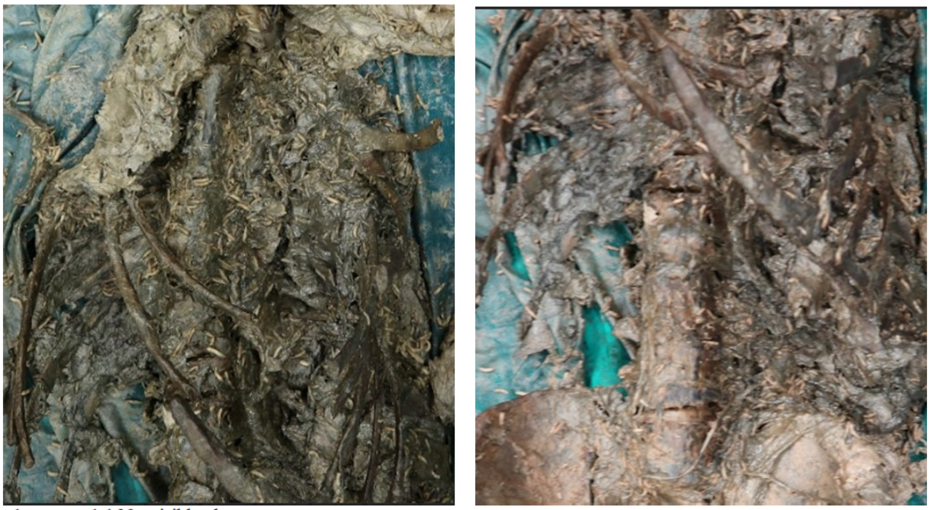

1 1.1 No visible changes
2 2.1 Livor mortis, rigour mortis and vibices
3 3.1 Grey to green discoloration
3.2 Bloating with green discoloration and/or skin blisters, skin slippage and/or marbling
3.3 Rectal purging of decompositional fluids
3.4 Post-bloating: release of abdominal gasses with discoloration changing from green to black
4 4.1 Decomposition of tissue producing sagging of flesh. Caving in of the abdominal cavity
4.2 Skin having a leathery appearance
4.3 Partial skeletonization, joints still together
5 5.1 Gross skeletonization, some joints disarticulated
6 6.1 Complete skeletonization

12. Which stage of decomposition does image no. 12 represent?

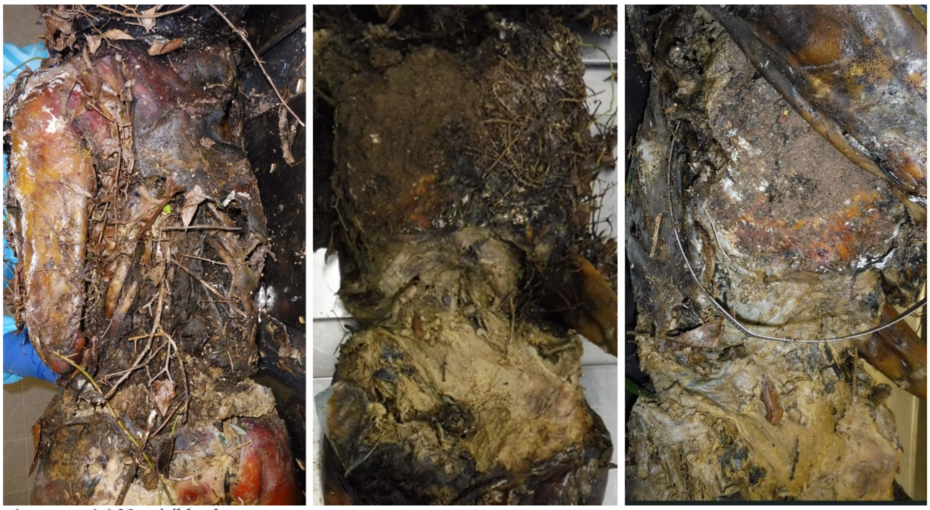

1 1.1 No visible changes
2 2.1 Livor mortis, rigour mortis and vibices
3 3.1 Grey to green discoloration
3.2 Bloating with green discoloration and/or skin blisters, skin slippage and/or marbling
3.3 Rectal purging of decompositional fluids
3.4 Post-bloating: release of abdominal gasses with discoloration changing from green to black
4 4.1 Decomposition of tissue producing sagging of flesh. Caving in of the abdominal cavity
4.2 Skin having a leathery appearance
4.3 Partial skeletonization, joints still together
5 5.1 Gross skeletonization, some joints disarticulated
6 6.1 Complete skeletonization

13. Which stage of decomposition does image no. 13 represent?

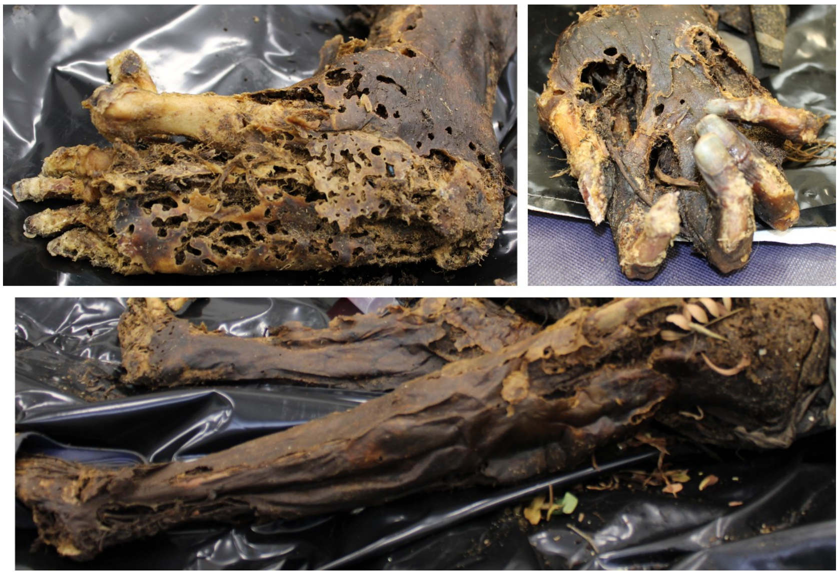

1	1.1 No visible changes
2	2.1 Livor mortis, rigour mortis and vibices
	2.2 Discoloration: brownish shades particularly at the edges. Drying of fingers and toes
3	3.1 Skin blisters and/or skin slippage and/or marbling
	3.2 Grey to green discoloration
	3.3 Brown to black discoloration
4	4.1 Skin having a leathery appearance
	4.2 Partial skeletonization, joints and tendons still together
5	5.1 Gross skeletonization, some joints disarticulated
6	6.1 Complete skeletonization

14. Which stage of decomposition does image no. 14 represent?

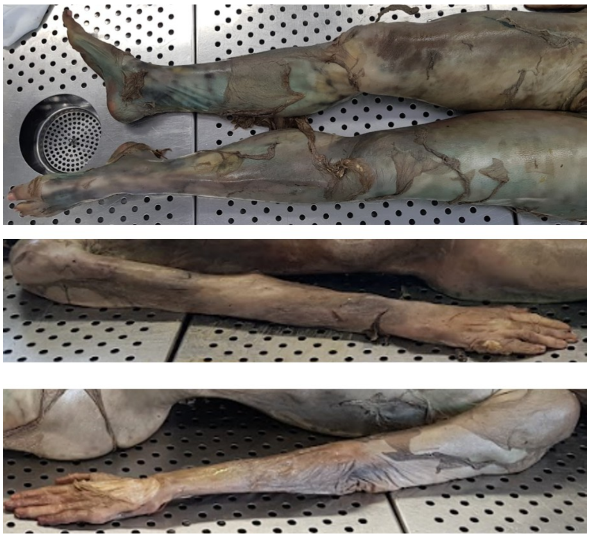

1	1.1 No visible changes
2	2.1 Livor mortis, rigour mortis and vibices
	2.2 Discoloration: brownish shades particularly at the edges. Drying of fingers and toes
3	3.1 Skin blisters and/or skin slippage and/or marbling
	3.2 Grey to green discoloration
	3.3 Brown to black discoloration
4	4.1 Skin having a leathery appearance
	4.2 Partial skeletonization, joints and tendons still together
5	5.1 Gross skeletonization, some joints disarticulated
6	6.1 Complete skeletonization

15. Which stage of decomposition does image no. 15 represent?

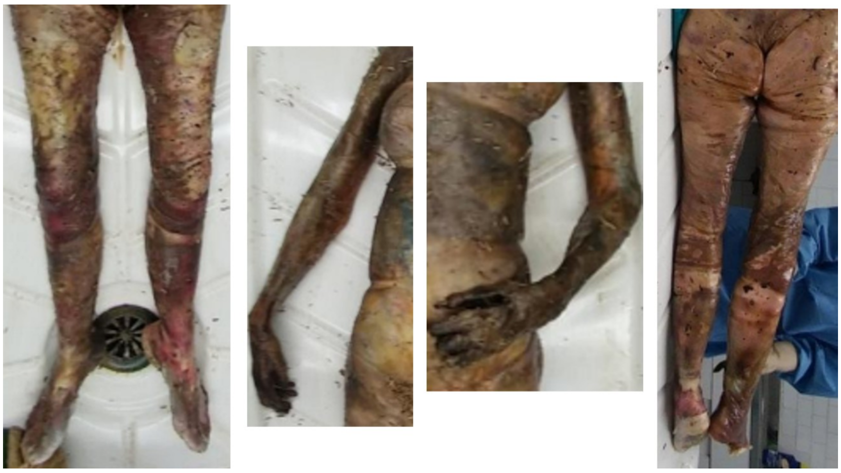

1	1.1 No visible changes
2	2.1 Livor mortis, rigour mortis and vibices
	2.2 Discoloration: brownish shades particularly at the edges. Drying of fingers and toes
3	3.1 Skin blisters and/or skin slippage and/or marbling
	3.2 Grey to green discoloration
	3.3 Brown to black discoloration
4	4.1 Skin having a leathery appearance
	4.2 Partial skeletonization, joints and tendons still together
5	5.1 Gross skeletonization, some joints disarticulated
6	6.1 Complete skeletonization

16. Which stage of decomposition does image no. 16 represent?

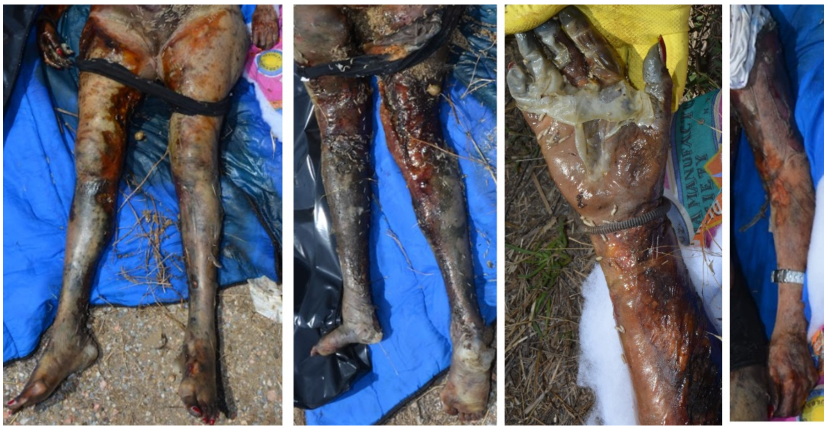

1	1.1 No visible changes
2	2.1 Livor mortis, rigour mortis and vibices
	2.2 Discoloration: brownish shades particularly at the edges. Drying of fingers and toes
3	3.1 Skin blisters and/or skin slippage and/or marbling
	3.2 Grey to green discoloration
	3.3 Brown to black discoloration
4	4.1 Skin having a leathery appearance
	4.2 Partial skeletonization, joints and tendons still together
5	5.1 Gross skeletonization, some joints disarticulated
6	6.1 Complete skeletonization

17. Which stage of decomposition does image no. 17 represent?

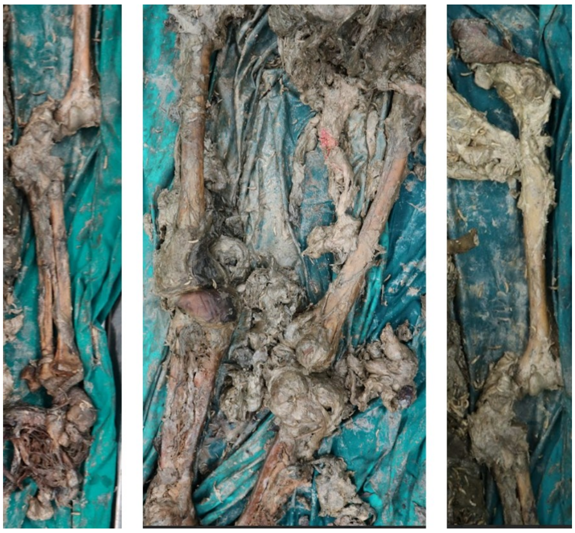

1	1.1 No visible changes
2	2.1 Livor mortis, rigour mortis and vibices
	2.2 Discoloration: brownish shades particularly at the edges. Drying of fingers and toes
3	3.1 Skin blisters and/or skin slippage and/or marbling
	3.2 Grey to green discoloration
	3.3 Brown to black discoloration
4	4.1 Skin having a leathery appearance
	4.2 Partial skeletonization, joints and tendons still together
5	5.1 Gross skeletonization, some joints disarticulated
6	6.1 Complete skeletonization

18. Which stage of decomposition does image no. 18 represent?

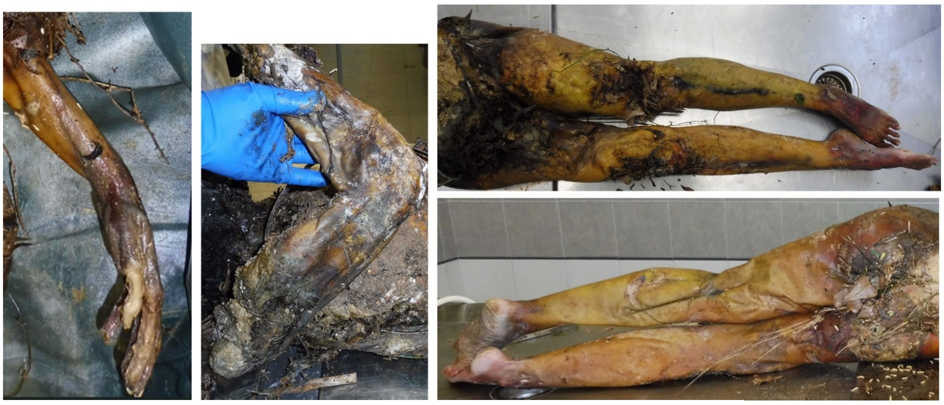

1	1.1 No visible changes
2	2.1 Livor mortis, rigour mortis and vibices
	2.2 Discoloration: brownish shades particularly at the edges. Drying of fingers and toes
3	3.1 Skin blisters and/or skin slippage and/or marbling
	3.2 Grey to green discoloration
	3.3 Brown to black discoloration
4	4.1 Skin having a leathery appearance
	4.2 Partial skeletonization, joints and tendons still together
5	5.1 Gross skeletonization, some joints disarticulated
6	6.1 Complete skeletonization
